# Supplementary material for: The Monomeric Conformational Ensembles of Aβ40 and Aβ42 Encode Their Differential Amyloid Aggregation Propensity
Source: J Phys Chem B. 2026 Jun 9;130(24):6035–46. doi: 10.1021/acs.jpcb.6c01000 (PMC13288636; doi:10.1021/acs.jpcb.6c01000)
Supplement: Supplementary file 1 [file jp6c01000_si_001.pdf]

Supporting Information
The Monomeric Conformational Ensembles of Aβ40 and Aβ42 Encode their Differential Amyloid Aggregation Propensity

Irene Cadenelli[1], Andrea Ciccolo[2], Andrea Tagliabue[1], Giulia Rossi[1], Valeria Conti Nibali[2,*], and Davide Bochicchio[1,*]

[1]*Department of Physics, University of Genoa, Genoa, 16146, Italy*
[2]*Department of Mathematical and Computational Sciences, Physical Sciences and Earth Sciences, University of Messina, Messina, 98166, Italy*
[*]*Corresponding authors: davide.bochicchio@unige.it, valeria.continibali1@unime.it*

1 Supporting

1.1 Convergence of Well-Tempered Metadynamics Simulations

Convergence of the WT-MetaD simulations [1] was assessed by monitoring the time evolution of a free energy difference between two selected regions of the one-dimensional FES projections along the biased collective variables n_α and n_β. The reported curves correspond to replica-averaged quantities obtained by combining the three independent simulations performed for each peptide (Fig. S1). For each CV, two windows were defined in the rescaled variables: a low-n region (0–5 residues), enriched in compact, weakly structured conformations, and a high-n region (5–30 residues), associated with more extended and highly structured states. The resulting $\Delta G(t)$ provides a practical and sensitive indicator of sampling stability in enhanced-sampling simulations of intrinsically disordered peptides and amyloid-β monomers [1, 2].

In a converging WT-MetaD simulation, $\Delta G(t)$ is expected to display damped oscillations and to approach a quasi-stationary regime as the accumulated bias becomes effectively time-independent. As shown in Fig. S1, both Aβ40 and Aβ42 exhibit decreasing oscillation amplitudes and fluctuations around slowly drifting mean values for both n_α and n_β. Residual long-time fluctuations are intrinsic to disordered systems, whose rugged and weakly funneled landscapes hinder strict numerical convergence on accessible simulation timescales, particularly for amyloid-β monomers [3, 4].

To ensure robust equilibrium estimates from reweighting, the initial 1 μs of each replica was excluded from all reweighted analyses, thereby removing the early transient regime characterized by rapid bias deposition. Reweighting was performed using standard WT-MetaD formalisms that reconstruct the equilibrium Boltzmann distribution from the biased dynamics [5]. After this equilibration interval, the overall FES topology and the relative stability of the low- and high-content *alpha* and β regions remain stable over extended time windows, supporting the reliability of the reweighted equilibrium ensemble.

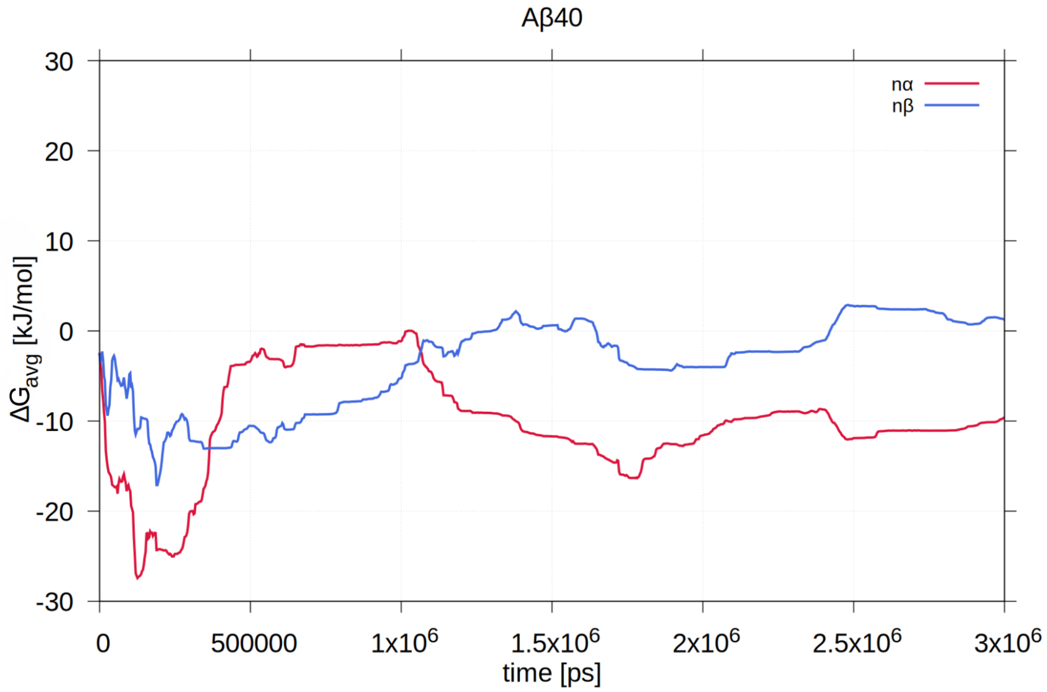

(a) Aβ40

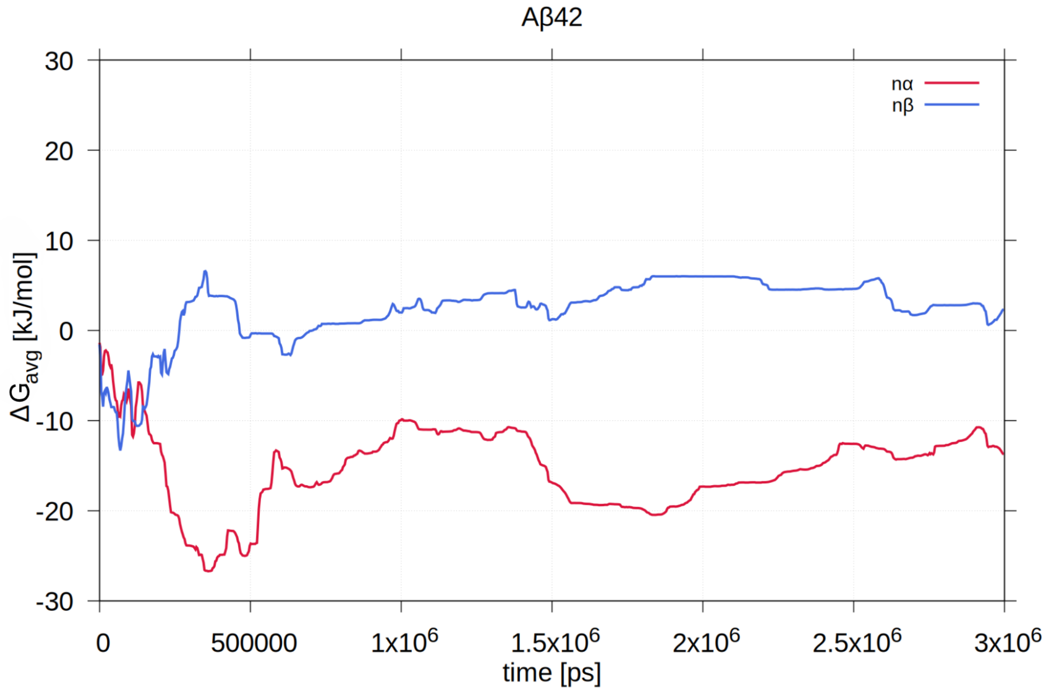

(b) Aβ42

Figure S1: Convergence of replica-averaged WT-MetaD simulations. Time evolution of the replica-averaged free energy difference ΔG_{avg} between low-n (0–5 residues) and high-n (5–30 residues) regions of the one-dimensional FES projections along n_α (red) and n_β (blue) for (a) Aβ40 and (b) Aβ42. Free-energy differences were estimated from WT-MetaD using standard estimators for converged bias potentials [1, 2].

1.2 Validation of the PLUMED-Based Secondary Structure Estimators

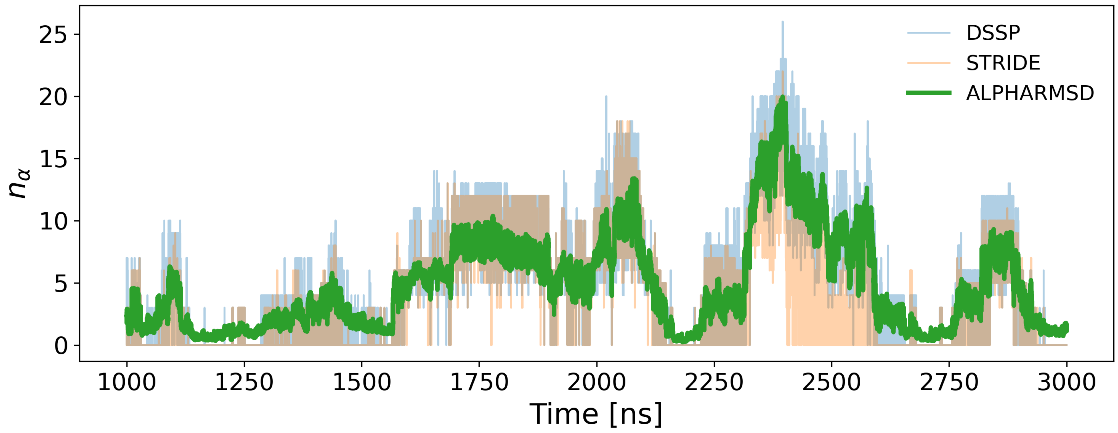

(a) Comparison of n_α estimates for Aβ42 (replica 1).

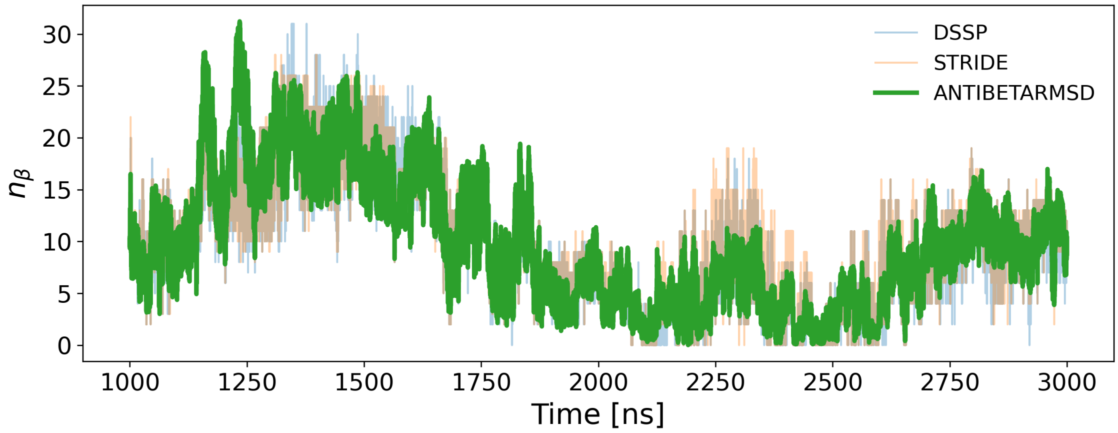

(b) Comparison of n_β estimates for Aβ42 (replica 1).

Figure S2: Validation of rescaled PLUMED collective variables for Aβ42. Time-series comparison between secondary structure content estimated using DSSP and STRIDE and the corresponding PLUMED CVs after rescaling. Panel (a) reports the number of residues in α-helical structure from DSSP, STRIDE, and the rescaled PLUMED variable ALPHARMSD ($n_\alpha = c_\alpha \cdot \text{ALPHARMSD}$). Panel (b) reports the analogous comparison for β-strand content using ANTIBETARMSD ($n_\beta = c_\beta \cdot \text{ANTIBETARMSD}$).

The PLUMED CVs `ALPHARMSD` and `ANTIBETARMSD` [6] provide continuous measures of α-helical and β-strand content, respectively, but are not directly interpretable as residue counts. To obtain physically meaningful residue-level estimates, we used established secondary structure assignment methods, namely DSSP [7] and STRIDE [8], which are routinely adopted as reference estimators for amyloid-β peptides and other IDPs [4, 9].

To establish a quantitative correspondence between PLUMED-derived observables and residue counts, multiplicative rescaling factors c_α and c_β were calibrated against DSSP and STRIDE assignments for Aβ42. For each of the three independent replicas, PLUMED-derived n_α and n_β were compared with the corresponding DSSP and STRIDE values over the 1.0–3.0 μs time interval, and optimal rescaling factors were determined by minimizing the least-squares deviation between the methods.

The resulting rescaling factors showed excellent consistency across replicas. Averaging over the three estimates yielded $c_\alpha = 1.1$ and $c_\beta = 2.5$. These values were subsequently used

throughout the study to convert PLUMED outputs into estimates of the number of residues participating in α-helical and β-strand structure. Representative time-series comparisons for replica 1 are shown in Fig. S2, demonstrating close agreement between the rescaled PLUMED variables and the DSSP and STRIDE references.

1.3 Complete Cluster Characterization

Figures S3–S6 provide a comprehensive cluster-resolved characterization of the conformational ensembles of Aβ40 and Aβ42 obtained from the consensus clustering analysis. Such a detailed description is particularly relevant for amyloid-β peptides, whose monomeric ensembles are intrinsically heterogeneous and span a wide range of structural and physicochemical states, even at equilibrium conditions [3, 4]. For each identified cluster, the reported tables include:

- Reweighted equilibrium population (%).
- Primary clustering descriptors (n_α, n_β, R_g, total SASA): weighted mean ± weighted standard deviation.
- Post hoc physicochemical descriptors (A-SASA, P-SASA, $n_{\text{HB-IP}}$, $n_{\text{HB-PS}}$): weighted mean ± weighted standard deviation.
- Global reference values (`ALL`): corresponding reweighted averages over the full unbiased ensemble.

Together, these data provide a complete numerical summary of all conformational families identified in the analysis. The cluster-resolved descriptors complement the qualitative discussion of the dominant conformational states reported in the main text and enable a direct quantitative comparison between Aβ40 and Aβ42 at the level of individual free-energy basins.

Rank	Relevance [%]	nα	nβ	Rg [nm]	SASA [nm²]
1	55.4	2.55 ± 1.48	9.65 ± 1.96	1.08 ± 0.0599	36.7 ± 1.89
2	11.8	1.95 ± 1.5	11.8 ± 2.4	1.3 ± 0.141	41.5 ± 1.67
3	8.3	3.38 ± 1.66	2.49 ± 1.63	1.3 ± 0.116	44.1 ± 1.81
4	7.09	1.86 ± 1.21	2.37 ± 1.82	1.61 ± 0.149	49.1 ± 2.14
5	6.92	4.18 ± 1.71	4.55 ± 1.72	1.14 ± 0.0918	39.2 ± 1.82
6	4.37	0.776 ± 0.588	9.45 ± 2.17	1.77 ± 0.216	47.2 ± 2.11
7	3.98	1.56 ± 1.7	1.75 ± 2.15	2.2 ± 0.273	53.1 ± 2.18
8	0.975	2.41 ± 1.45	18.2 ± 3.62	1.1 ± 0.101	35.2 ± 1.98
9	0.796	9.19 ± 1.51	2.53 ± 1.51	1.2 ± 0.0969	40.2 ± 2.07
10	0.222	8.54 ± 2.2	0.849 ± 0.91	1.59 ± 0.185	47 ± 2.12
11	0.0768	8.49 ± 1.47	7.67 ± 2.15	1.08 ± 0.0816	36 ± 1.71
12	0.0213	13.9 ± 1.66	2.83 ± 1.61	1.07 ± 0.0838	36.4 ± 1.77
13	0.00373	15.5 ± 1.43	1.76 ± 1.47	1.25 ± 0.126	39.8 ± 1.89
14	0.000469	12.9 ± 1.6	6.83 ± 1.88	0.984 ± 0.0414	33.2 ± 1.32
15	0.000405	9.13 ± 1.9	12.7 ± 2.29	1.34 ± 0.138	39.9 ± 1.65
16	0.000227	17.9 ± 2.84	0.822 ± 0.607	1.5 ± 0.137	43.8 ± 1.86
17	0.000166	19.7 ± 2	1.48 ± 0.963	1.12 ± 0.135	36.2 ± 2.17
ALL		6.27 ± 5.3	7.39 ± 6.02	1.21 ± 0.256	39 ± 4.82

Figure S3: Cluster-resolved structural descriptors for Aβ40. Reweighted equilibrium populations and weighted averages of n_α, n_β, radius of gyration R_g, and total SASA for all consensus clusters.

Rank	Relevance [%]	nα	nβ	Rg [nm]	SASA [nm²]
1	56.2	2.74 ± 1.75	12 ± 2.17	1.14 ± 0.0749	38.1 ± 2.03
2	11.3	2.89 ± 1.64	2.85 ± 1.85	1.42 ± 0.142	46 ± 2.25
3	9.71	0.936 ± 0.602	7.58 ± 2.11	1.57 ± 0.205	47.5 ± 2.3
4	8.12	3.87 ± 1.57	5.17 ± 2.24	1.17 ± 0.0945	40.7 ± 2.11
5	6.95	1.25 ± 1.08	1.44 ± 1.77	2.03 ± 0.307	54.6 ± 2.6
6	4.46	4.2 ± 1.91	10.8 ± 2.47	1.47 ± 0.126	43.6 ± 1.4
7	2.47	1.61 ± 1.33	16.5 ± 2.39	1.51 ± 0.171	42.7 ± 1.61
8	0.499	2.36 ± 1.55	20.5 ± 3.18	1.18 ± 0.0868	36.7 ± 1.66
9	0.194	7.14 ± 1.52	6.49 ± 3.06	1.06 ± 0.0619	36.5 ± 2.19
10	0.0699	5.84 ± 2.51	0.446 ± 0.888	2.29 ± 0.338	54.6 ± 2.67
11	0.00967	10.7 ± 1.9	3.02 ± 2.18	1.14 ± 0.109	39.3 ± 3.22
12	0.000699	11.4 ± 2.15	1.2 ± 1.44	1.46 ± 0.154	46.9 ± 2.15
13	5.32e-08	15.3 ± 2.14	4.05 ± 2.6	1.38 ± 0.14	42.7 ± 2.22
ALL		4.6 ± 3.42	8.59 ± 6.56	1.26 ± 0.277	40.6 ± 5.18

Figure S4: Cluster-resolved structural descriptors for Aβ42. Same quantities as in Fig. S3, reported for the Aβ42 conformational ensemble.

Rank	Relevance [%]	A-SASA [nm²]	P-SASA [nm²]	nHB-IP	nHB-PS
1	55.4	14 ± 1.74	20.5 ± 2.02	15.5 ± 3.35	123 ± 8.14
2	11.8	16.5 ± 1.85	22.2 ± 1.81	14.3 ± 2.74	128 ± 7.03
3	8.3	18 ± 1.72	23 ± 1.72	10 ± 3.01	136 ± 7.68
4	7.09	20.4 ± 1.84	25.2 ± 1.72	7.45 ± 2.8	143 ± 7.63
5	6.92	15.4 ± 1.9	21.3 ± 1.92	12.8 ± 3.01	128 ± 7.47
6	4.37	19.6 ± 1.61	24.4 ± 1.92	10.1 ± 2.81	139 ± 7.66
7	3.98	22.6 ± 1.71	27 ± 1.75	5.72 ± 2.41	148 ± 7.4
8	0.975	13.1 ± 1.49	19.9 ± 1.49	18.9 ± 3.15	116 ± 7.34
9	0.796	15.6 ± 1.77	22.1 ± 1.72	14.6 ± 3.17	125 ± 7.45
10	0.222	19.1 ± 1.59	24.6 ± 1.6	11.6 ± 2.97	133 ± 7.27
11	0.0768	13.9 ± 1.57	20.1 ± 1.69	17.9 ± 3.2	117 ± 7.3
12	0.0213	13.4 ± 1.89	20.5 ± 1.59	18.6 ± 3.4	116 ± 7.18
13	0.00373	15.1 ± 1.16	22.2 ± 1.35	17.9 ± 3.28	119 ± 7.52
14	0.000469	12.4 ± 1.77	18.4 ± 1.47	21.1 ± 3.32	110 ± 6.83
15	0.000405	14.9 ± 1.32	22.8 ± 1.26	18.5 ± 2.98	119 ± 6.53
16	0.000227	17.4 ± 1.46	23.6 ± 1.63	17.9 ± 3.63	121 ± 8.14
17	0.000166	13.7 ± 1.26	20.3 ± 1.44	21.3 ± 3.08	110 ± 6.87
ALL		15.2 ± 2.88	21.4 ± 2.46	15.4 ± 4.96	124 ± 11.8

Figure S5: Extended physicochemical descriptors for Aβ40. Cluster-resolved apolar and polar SASA and hydrogen-bond metrics (nHB-IP and nHB-PS).

Rank	Relevance [%]	A-SASA [nm²]	P-SASA [nm²]	nHB-IP	nHB-PS
1	56.2	14.1 ± 1.67	21.5 ± 1.31	16.4 ± 3.29	125 ± 8.04
2	11.3	18.9 ± 1.65	24.2 ± 1.52	10 ± 2.83	140 ± 7.55
3	9.71	19.3 ± 1.49	25.2 ± 1.75	10.3 ± 2.72	141 ± 7.62
4	8.12	15.7 ± 1.84	22.3 ± 1.48	13.5 ± 2.89	131 ± 7.38
5	6.95	23.6 ± 2.1	27.3 ± 1.36	5.58 ± 2.48	152 ± 7.61
6	4.46	18 ± 1.13	23.2 ± 1.21	14.3 ± 2.96	132 ± 7.18
7	2.47	17.2 ± 0.971	22.9 ± 1.41	15.5 ± 2.74	131 ± 7.2
8	0.499	14 ± 1.79	20.2 ± 1.49	20.3 ± 3.05	118 ± 7.66
9	0.194	13.1 ± 1.73	20.9 ± 1.32	18 ± 3.33	120 ± 7.67
10	0.0699	25 ± 1.38	26 ± 1.5	7.84 ± 3.31	148 ± 8.54
11	0.00967	15.8 ± 2.23	21.1 ± 1.78	17 ± 4	123 ± 9.19
12	0.000699	20.3 ± 1.67	23.7 ± 1.3	13.6 ± 3.07	133 ± 7.07
13	5.32e-08	17.8 ± 1.38	22.6 ± 1.35	19.2 ± 2.83	123 ± 6.81
ALL		15.9 ± 3.28	22.1 ± 2.26	15.4 ± 4.89	128 ± 11.9

Figure S6: Extended physicochemical descriptors for Aβ42. Same quantities as in Fig. S5, reported for the Aβ42 conformational ensemble.

1.4 Per-replica secondary-structure probabilities

To assess the reproducibility of the residue-level secondary-structure analysis across independent simulations, we computed the per-residue secondary-structure probabilities separately for each replica, using the same analysis protocol adopted for the averaged profiles reported in the main manuscript.

Figure S7 reports the per-replica secondary-structure probabilities for both Aβ40 and Aβ42. Overall, the main residue-specific trends observed in the replica-averaged profiles are preserved across the independent trajectories. Local differences in probability values are present, as expected for intrinsically disordered peptides, but no marked qualitative discrepancy between replicas is observed. These results support the robustness of the secondary-structure features discussed in the main text.

1.5 Per-replica intra-peptide contact maps

To further evaluate the consistency of the structural ensembles sampled by the independent simulations, we also computed the intra-peptide contact maps separately for each replica. The same contact definition and analysis protocol used for the replica-averaged maps reported in the main manuscript were applied.

The per-replica contact maps are shown in Figure S8. The dominant contact patterns are consistently recovered across replicas for both peptides. As expected, some replica-dependent variations are observed in the intensity of individual contacts, reflecting the conformational heterogeneity of the monomeric ensembles. However, the overall topology of the contact maps remains comparable among independent trajectories, supporting the reproducibility of the intra-peptide interaction patterns discussed in the main text.

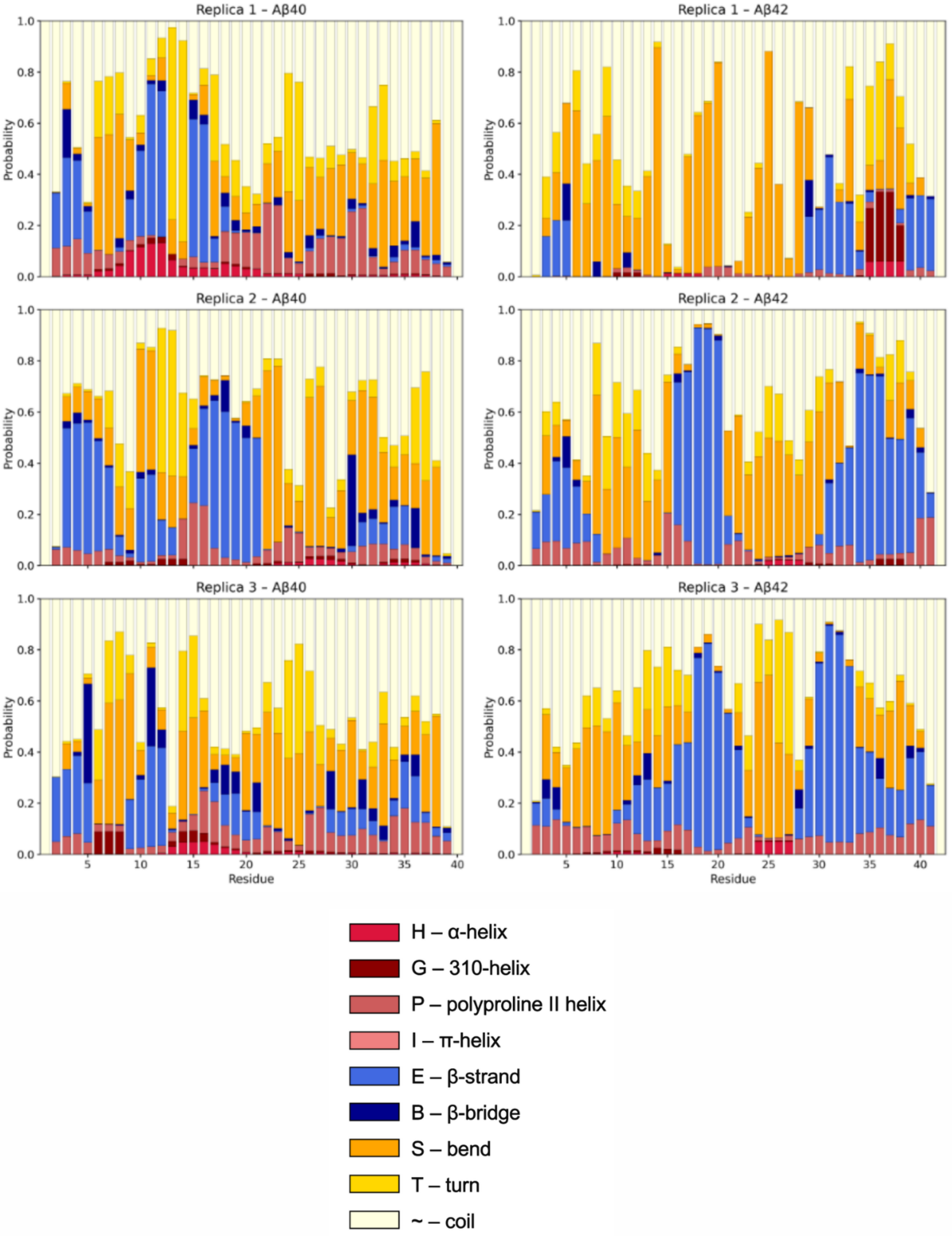

Figure S7: Per-replica per-residue secondary-structure probabilities for Aβ40 and Aβ42.

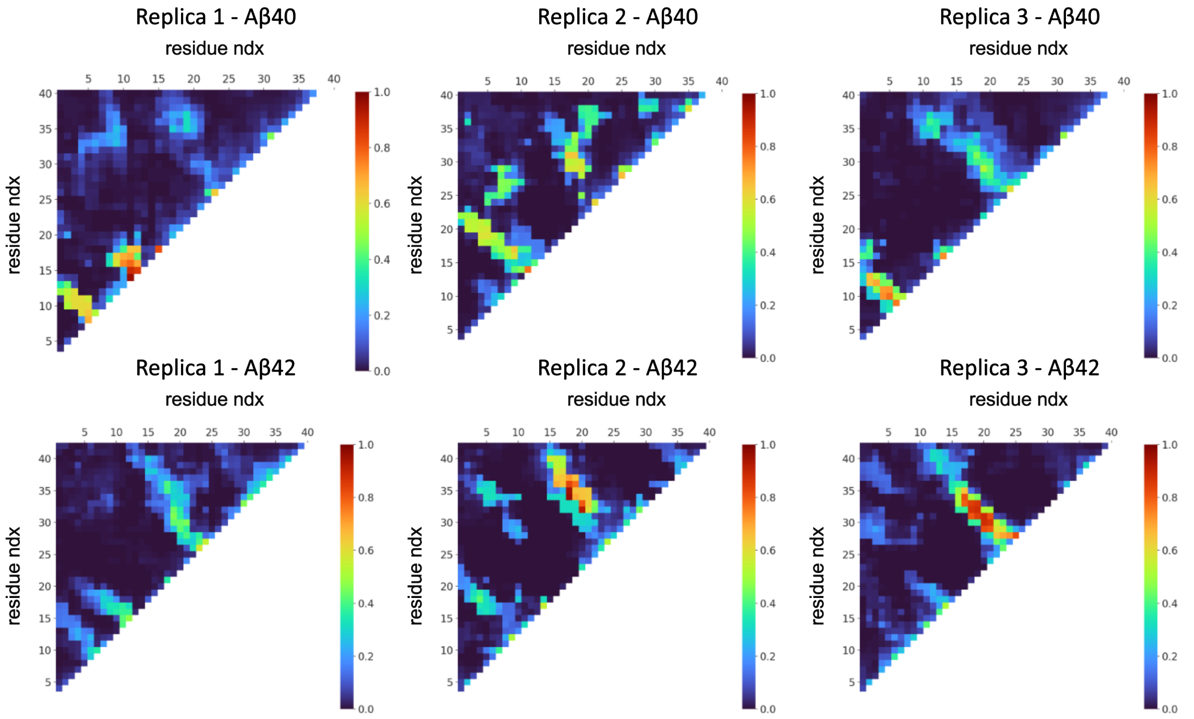

Figure S8: Per-replica intra-peptide contact probability maps for Aβ40 and Aβ42.

References

(1) Barducci, A.; Bussi, G.; Parrinello, M. *Physical Review Letters* **2008**, *100*, DOI: `10.1103/physrevlett.100.020603`.

(2) Tiwary, P.; Parrinello, M. *The Journal of Physical Chemistry B* **2014**, *119*, 736–742.

(3) Chakraborty, D.; Straub, J. E.; Thirumalai, D. *Science Advances* **2023**, *9*, DOI: `10.1126/sciadv.add6921`.

(4) Nguyen, P. H.; Ramamoorthy, A.; Sahoo, B. R.; Zheng, J.; Faller, P.; Straub, J. E.; Dominguez, L.; Shea, J.-E.; Dokholyan, N. V.; De Simone, A.; Ma, B.; Nussinov, R.; Najafi, S.; Ngo, S. T.; Loquet, A.; Chiricotto, M.; Ganguly, P.; McCarty, J.; Li, M. S.; Hall, C.; Wang, Y.; Miller, Y.; Melchionna, S.; Habenstein, B.; Timr, S.; Chen, J.; Hnath, B.; Strodel, B.; Kayed, R.; Lesné, S.; Wei, G.; Sterpone, F.; Doig, A. J.; Derreumaux, P. *Chemical Reviews* **2021**, *121*, 2545–2647.

(5) Bonomi, M.; Barducci, A.; Parrinello, M. *Journal of Computational Chemistry* **2009**, *30*, 1615–1621.

(6) Pietrucci, F.; Laio, A. *Journal of Chemical Theory and Computation* **2009**, *5*, 2197–2201.

(7) Kabsch, W.; Sander, C. *Biopolymers* **1983**, *22*, 2577–2637.

(8) Heinig, M.; Frishman, D. *Nucleic Acids Research* **2004**, *32*, W500–W502.

(9) Qiu, T.; Liu, Q.; Chen, Y.-X.; Zhao, Y.-F.; Li, Y.-M. *Journal of Peptide Science* **2015**, *21*, 522–529.
